# Milk-Sickness

**Published:** 1837

**Authors:** 


### Art. III.—Milk Sickness.

In the same Journal we find another Inaugural thesis, by Dr. J. Newton Smith, one of our esteemed pupils of former years, on the disease called by the people of the west, "MILK SICKNESS," or "SICK STOMACH." Dr. Smith has adopted the opinion that it orinates from a poisonous plant, and offers, in support of this hypothesis, the following facts:

As to its vegetable origin, we do not think there is so much uncertainty. Circumstances having placed us, for the last four years, in a part of the country particularly harassed by the frequent occurrence of this disease, we have taken a great deal of pains to ascertain, as nearly as possible, its nature and causes.

That the disease is produced by using the milk, or meat, of such cattle as run at large in our woods, is certain. And that they have taken it, from using certain vegetable productions, may be fairly inferred from the following facts:

1st. You may fence in a piece of ground in its native state, and turn upon it a lot of cattle, and they will all take the disease. Then go into it with your axes and hoes, thin it out, and dig up every weed, with the nature and properties of which you are ignorant, and destroy them. And you may, with confidence, turn in your milch cows, and they will not take the disease;—or,

2d. You may take advantage of a dry time in autumn, and burn the woods, and repeat it for two or three years in succession, and you may turn your stock upon it without fear.

About fifteen or twenty years ago, we are told that this disease was particularly severe in the edge of an adjoining county, and from some accident during a dry time in autumn, the woods became ignited, and burned over a circumference of several miles;—and in the following autumn, either by accident or design, the burning was repeated. Since which time, there has never been a case of the disease in that neighborhood. And the inhabitants do not even think it necessary to keep up their cattle.

3d. You may clear out a piece of ground and attend it for two or three years in grain, or grass of any description, when your stock may go on it with perfect safety.

We know a gentleman of respectability, who lives, and has lived for the last thirty or forty years, in the very heart of one of those infested districts, and whose family and stock, some years ago, suffered from repeated attacks of this disease. He, believing that it was produced by some vegetable that was very abundant on his place, and seeing that its effects disappeared on clearing up the grounds, and exposing them to the action of the sun, commenced the plan of clearing a piece of ground every year; that is, thinning out, or cutting out the smaller trees, raking up and burning the trash, and sowing it in blue grass and timothy. After it is well set with grass, he invariably turns on it his stock, with perfect safety. The blue grass having a tendency to root out and destroy any other vegetable, when they happen to meet on the same ground. This gentleman has now several hundred acres in grass, on which he raises a great deal of stock for the Cincinnati market; and my own family often bought butter produced from cows running on the same grounds; which, a few years previously, were so abundantly supplied with the cause of the milk sickness as to produce the disease, in cattle feeding on it but a few hours.

The lands adjoining these pastures are yet as bad as ever. Thus you see, on one side of the fence, cattle feeding in perfect security—turn them on the other side, but for a few hours, at the proper season of the year, and they are very soon the subjects of the milk sickness. It is not considered dangerous to let cattle run out until the latter part of summer, when vegetables have produced their seed. And most of the cases occur at, and after the time of the first hard frosts.

As a means of diagnosis the Doctor makes the following recommendations:

If we are yet in doubt, as to the nature of the disease under which our patient labors, we may direct a pint, or a quart of the suspected milk to be given to a dog—and, if the poison is present, the fine, playful, spirited fellow will soon manifest it—he soon retires, trembling, to some quiet place of rest; and the fierce animal, that a few hours before had opposed and rendered our entrance upon his domain a matter of great hazard, will now suffer us, were we his worst enemies, to approach without notice.

Those who are much afraid of taking the disease, have many ways of detecting its presence in their cows. If the cow is laboring under it, the calf will frequently suck until it falls to the ground—and in most cases it is first discovered in the calf.

There is a more certain way of guarding against the effects of this poison; it is a most common practice, with many families in our neighborhood, to boil all their milk before using it; and they have assured us, that they can, with certainty, detect the presence of this deleterious agent, at all times, by the appearance of the milk when heated, They say that it invariably coagulates, curdles, immediately on being heated, when the cow has the milk disease. We know one family in particular, that has escaped the disease year after year, by this simple precaution, although it has been a very common thing with them to lose their stock.

The following is his statement of the plan of cure in this malady, which does not materially differ from that recommended by other gentlemen, resident in tracts of country where it is endemic.

Treatment.—The first stage of this disease requires very little medicine, and is very easily relieved. The only things necessary, are a state of perfect quietude, and some of the milder laxatives, as the ol. ricini or sulphate magnesia, given so as to keep up a loose state of the bowels, or an emetic of ipecacuanha, to be followed up by these laxatives.

The second state requires a much more energetic practice.

And from the character of the symptoms, we would be led, at once, to the use of depletory remedies. Bleeding, in the commencement of this stage, would naturally be suggested, as a remedy of great value. Yet we have never, in one single instance, had a patient, who would submit to venesection, in consequence of a belief, in our part of the country, that this operation is invariably succeeded by death. How this belief originated, we know not, but we are convinced that, so far as it prevails, it has robbed the profession of a valuable agent.

We have had the care of a great many cases, and have been favored with very uniform success, whenever we have been permitted to have our own way.

Purging seems to be an all important remedy. We have usually given the calomel, in small and frequently repeated doses. After repeating them until the gastric disturbance is, in some degree allayed, we have generally resorted to some auxiliary, to promote their operation, as the ol. ricini, sulphate mag. or the latter, in combination with senna tea. These rarely fail to bring off copious feculent alvine discharges, which pretty generally alter the character of the symptoms, for the better. The balance of the circulation is restored—the pulse is improved—the skin feels more natural, and the countenance loses, in some degree, its unnatural, cadaverous appearance.

By the daily repetition of the calomel, &c. the secretions of the liver are unlocked. The chylopoietic viscera assume their wonted action, and a very few days will now suffice to place the patient out of danger. When he is very soon restored to health, by the occasional use of the pills of rheubarb, aloes and soap.

The greatest difficulty to be encountered, in this stage, is the constant tendency to vomit. We have often allayed this by mustard poultices over the region of the stomach, and by the use of the saline mixture; when the gastric irritability is very great, blisters are indispensable.

Others have pursued a different practice, and with equal success. In the first place, they administer an emetic, in such a manner as to vomit only about once or twice; when they give something to throw its action upon the bowels. They rely, solely, upon the sulph. mag., as being particularly suitable to this disease; being retained on the stomach, often, when other articles are rejected. We have seen many relieve themselves, simply by this article frequently repeated.

But all these means should be used promptly, or a very few hours may place the patient at the threshold of the third stage, where all remedies are alike useless.

From the foregoing facts we may draw the following inferences:—1st. That the milk sickness is produced by a vegetable poison. 2d. That this poison, diffusing itself throughout the system, produces disorder of all the functions of the body, and that, thus far, it is a disease that is under the control of remedial agents. And 3d, when this functional becomes structural disorder, it ceases to be manageable. D.

